# A Novel Semi-Analytical Model for Multi-branched Fractures in Naturally Fractured-Vuggy Reservoirs

**DOI:** 10.1038/s41598-018-30097-2

**Published:** 2018-08-02

**Authors:** Lei Wang, Xiaoxia Chen, Zunyi Xia

**Affiliations:** 1 ERE & BIC-ESAT, College of Engineering, Peking University, Beijing, 100871 China; 2 School of Earth Sciences and Resources, China University of Geosciences, Beijing, 100083 China

## Abstract

In the past few decades, scholars have made great breakthroughs in the study of well test analysis of carbonate rock. The previous studies are based on horizontal wells, straight wells, fractured wells, and inclined wells. With the development of fracturing technology, acid fracturing technology is considered to be the most effective measure to develop carbonate reservoirs. As the carbonate rock is easily dissolved in carbonic acid, multi-branched fractures will be produced near a vertical well. This article presented a semi-analytical model for multi-branched fractures in naturally fractured-vuggy reservoirs for the first time, which laid a theoretical foundation for solving well test analysis for finite conductivity multi-branched fractures. The model can quantify the wellbore flow pressure and applied to obtain more parameters reflecting comprehensive flow characteristics through using history matching procedure. The results were compared with numerical simulation and the existing analytical solutions of a single fracture model. Then in this paper, flow characteristics are recognized and there are five flow regimes found in the type curves, e.g. bi-linear flow region, linear flow region, inter-porosity flow region between vugs and fractures, inter-porosity region between matrix and fractures, and radial flow region. Finally, the influence factors analysis shows fracture number will mainly affect flow behavior of bi-linear flow and linear flow. The angle analysis showed that as the fractures were closer, their interaction became stronger. The conductivity would seriously affect the flow behavior in the early time. Linear flow cannot be observed when the conductivity is less than 1 and bi-linear flow cannot be observed when the conductivity is more than 20. And the effect of fracture length on flow behavior occurs in the early time. Bi-linear flow and linear flow characteristics cannot be observed when the fracture length is reduced.

## Introduction

Fractured-vuggy rock with complex pore structures has been widely studied by many countries^1–9^. Well test analysis in hydraulically fractured wells has been a topic of interest to the oil industry since early 1950s. Classic analytical and semi-analytical models have been developed for many years. In 1973, Gringarten and Ramey^10^ first introduced source functions for transient pressure analysis of uniform flux fractures and infinite conductivity fracture wells. Taking account for more realistic cases, Cinco-Ley *et al*.^11^ extended the Green’s functions to the transient pressure solutions. Cinco-Ley and Samaniego^12^ also proposed a bi-linear flow model for analyzing early-time pressure data and type curves for identifying all the flow regimes for wells intersected by finite-conductivity fractures. Furthermore, general solutions considering the effects of dual-porosity effect in the reservoir was presented by Cinco-Ley and Meng^13^. The above solutions become the basic solutions of production data analysis^14–17^ and some authors also considered fracture asymmetry based on the basic solutions^18–22^.

**Figure 1:**
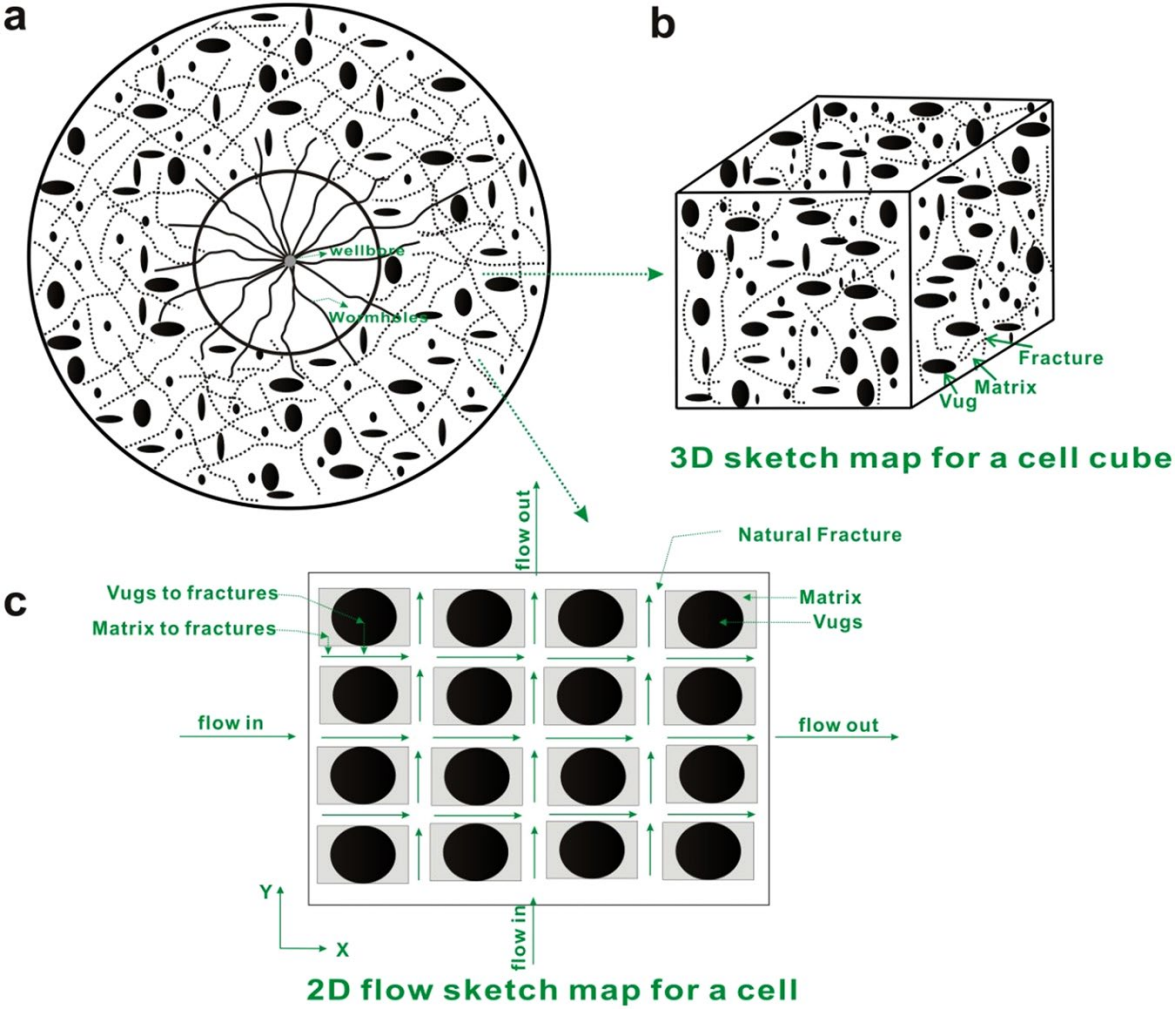
Schematic diagram: (**a**). Fractured-vuggy carbonate reservoirs with multi-branched fractures. (**b**) the system of matrix, fractures, vugs. (**c**) 2D flow sketch map for a cell (Wang *et al*. 2014).

However, only a single fracture around a vertical well was considered in the above models^10–13,18–22^. Carbonate minerals are easy to be dissolved by carbonic acid^23,24^, which is often used to react with the rock to create a high channel as possible, namely wormhole (Fig. 1), and this process is called as carbonate acidizing. These wormholes are similar to multiple fractures systems from stimulated reservoir volume (SRV)^25^. Dora Patricia Restrepo in his ph.D’s dissertation^25^ investigated the pressure behavior of a system containing near wellbore and far field multiple fractures. He also extended the model into dual-porosity media. However, only infinite conductivity model was investigated so that some phenomenon in early time couldn’t be observed.

Fractured-vuggy reservoirs are generally composed of matrix, fractures and vugs systems with varying properties. Generally speaking, both porosity and permeability are different from those systems^9,26^. Both analytical and numerical methods can be used to simulate the flow behaviors in these systems^27–37^. However, pressure transient behavior for multi-branched fractures has been rarely reported in previous literatures.

In 2014, Wang *et al*.^34^ presented a multi-branched model with an infinite conductivity, however, the flow in the fractures was not considered in their model so that the regime in the early time was not observed. In this paper, a finite conductivity multi-branched fractures model for carbonate reservoirs was studied in details. We focused on a single phase transient flow behavior in fractured-vuggy rock, conceptualized as multiple-continuum medium, consisting permeable natural fractures, low-permeability rock matrix, vugs, and multi-branched fractures. It was assumed that vugs which provided storage space were dispersed throughout fractured carbonate reservoirs, and the fractures were responsible for global flow. Use of analytical techniques, a Laplace-transform model is established and the semi-analytical solutions are successfully derived. The solutions are presented and compared with numerical simulation and the existing analytical solutions of a single fracture model. The presented model can be used to analyze the well test data and production date through using history matching procedure in the future.

## Mathematical Models

### Basic assumptions

**Figure 2:**
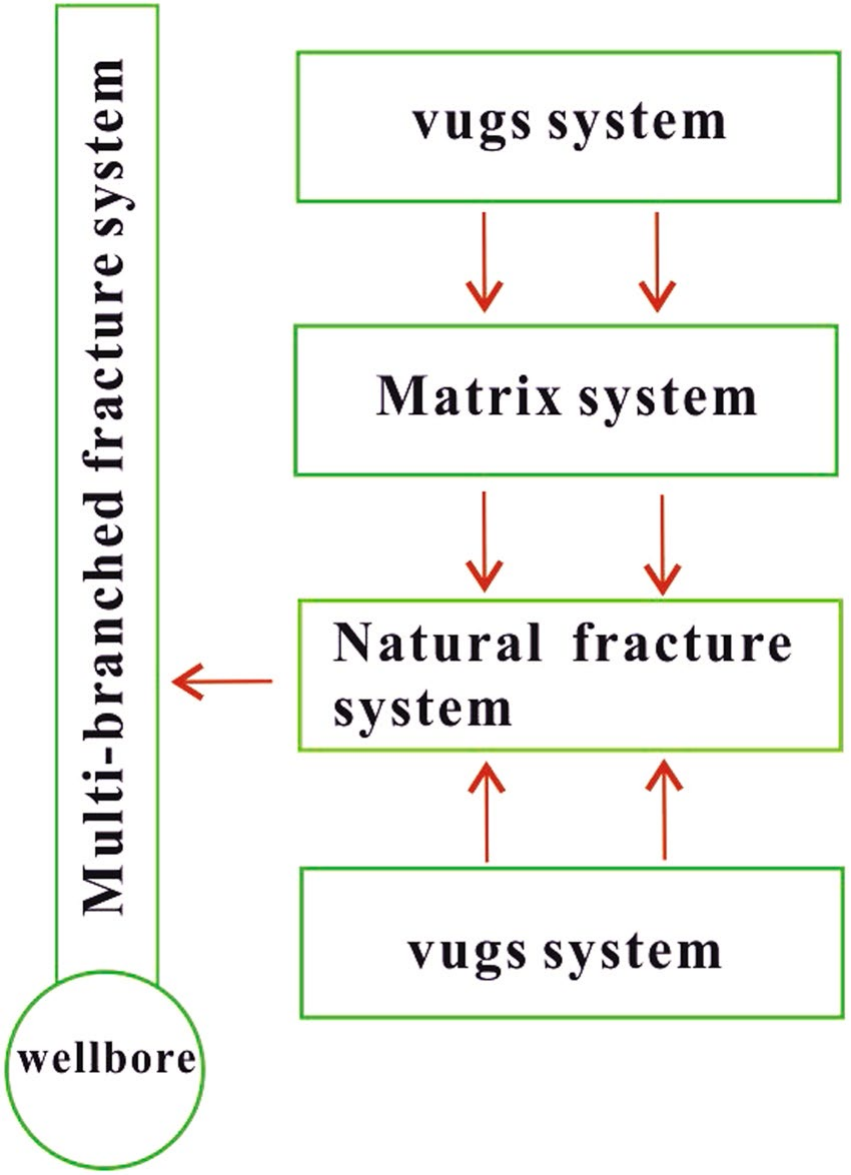
Physical modeling sketch map of fractured-vuggy carbonate medium.

Most of the researchers consider the non-Darcy effect during the process of gas seepage near the wellbore because of the high velocity of gas flow^38–44^. However, they also think that although non-Darcy flow may occur, the assumption of Darcy (linear) flow for oil at near the wellbore is reasonable because of relatively low velocity of oil flow. Fractured-vuggy carbonate reservoirs with multi-branched fractures are naturally structured by matrix system, natural fractures system, vugs system. (See Fig. 1a,b). Figure 2 shows that the physical modeling scheme of fractured-vuggy carbonate medium. The assumptions of physical model are as follows:

1. Sugar cube fractures as seen in classical multiple porosity media models are used in this article^4,5,37^; shape factors of fracture-vug, fracture-matrix, and vug-matrix could be defined as *α*_*fv*_, *α*_*fm*_, and *α*_*vm*_^5,29^, respectively.
2. The reservoir permeability, porosity, compressibility, are constants.
3. Isothermal process and Darcy flow are assumed.
4. The total production of multi-branched fractures is equal to total production Q.
5. The well produces with constant-viscosity and slightly compressible fluid, but the flux of each fracture changes with the time *t*.
6. All fractures length may be unequal and the fractures are assumed to be finite conductivity.
7. The initial pressure is equal to *p*_*i*_.

### Reservoir Model

Some reservoir models for vertical wells in carbonate reservoirs have been studied^4,5,23,34^. Figure 1c shows the flow process. Derivation and description of details for reservoir model can be found in **Appendix B** of Supplementary material. Through using the superposition principle and double Fourier transform, the dimensionless pressure of Laplace domain is given by

1 $${\tilde{p}}_{fD}({x}_{D},{y}_{D},s)=\sum _{n=1}^{{N}_{f}}{\int }_{0}^{{L}_{fDn}}\frac{1}{{L}_{fDn}}{\tilde{q}}_{fDn}({u}_{Dn},s)F({x}_{D},{y}_{D},{x}_{fDn},{y}_{fDn},{\theta }_{fn},s)d{u}_{Dn}$$

2 $$\begin{array}{c}F({x}_{D},{y}_{D},{x}_{fDn},{y}_{fDn},{\theta }_{fn},s)\\ \,={K}_{0}[\sqrt{sg(s)}\sqrt{{({x}_{D}-{x}_{fDn}-{u}_{Dn}\cos {\theta }_{fn})}^{2}+{({y}_{D}-{y}_{fDn}-{u}_{Dn}\sin {\theta }_{fn})}^{2}}]\end{array}$$

3 $$g(s)={w}_{f}+\frac{({\lambda }_{fv}+{\lambda }_{fm})s+\frac{1-{\omega }_{f}}{{\omega }_{v}{\omega }_{m}}[{\lambda }_{fv}{\lambda }_{fm}+({\lambda }_{fv}+{\lambda }_{fm}){\lambda }_{vm}]}{{s}^{2}+[\frac{{\lambda }_{fv}}{{\omega }_{v}}+\frac{{\lambda }_{fm}}{{\omega }_{m}}+(\frac{1}{{\omega }_{v}}+\frac{1}{{\omega }_{m}}){\lambda }_{vm}]s+\frac{{\lambda }_{fv}{\lambda }_{fm}+({\lambda }_{fv}+{\lambda }_{fm}){\lambda }_{vm}}{{\omega }_{v}{\omega }_{m}}}$$

Where ${\tilde{p}}_{fD}$ is the dimensionless fracture pressure of Laplace domain; ${\tilde{q}}_{fD}$ is dimensionless fracture flow rate of Laplace domain; (*x*_*D*_, *y*_*D*_) is dimensionless coordinates. (*x*_*fDn*_, *y*_*fDn*_) are the coordinates of the starting point for sources of *n*th fracture; *θ*_*fn*_ is the angle between the *n*th fracture and *x* axis; *u*_*Dn*_ is point source position of *n*th fracture, integral variable; *L*_*fDn*_ is dimensionless length of *n*th fracture; *N*_*f*_ is fracture number; *s* is dimensionless time variable of Laplace domain; *ω*_*f*_ is fracture storage coefficient; *ω*_*v*_ is vug storage coefficient; *ω*_*m*_ is matrix storage coefficient; *λ*_*fv*_ is fracture-vug inter-porosity flow coefficient; *λ*_*fm*_ is fracture-matrix inter-porosity flow coefficient; *λ*_*vm*_ is vug-matrix inter-porosity flow coefficient; *K*_0_(x) is the modified Bessel function (2nd kind, 0 order).

### Fracture Flow Model

**Figure 3:**
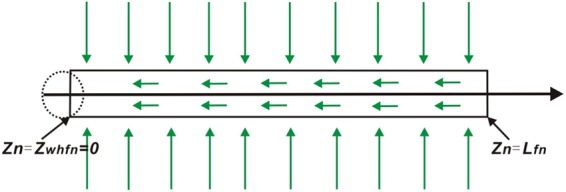
Linear flow in the fractures.

The well is fixed in the endpoint. Linear flow occurs in the fractures (Fig. 3). Establishment and solution of fracture flow model can be found in **Appendix C** of Supplementary material. The pressure drop at *k*th segment on the nth fracture is given by

4 $${\tilde{p}}_{fDnk}({z}_{Dnk},s)={\tilde{p}}_{fDn}(0,s)+\frac{2\pi }{{L}_{fDn}^{2}{C}_{fDn}}{R}_{1}-\frac{2\pi }{{L}_{fDn}{C}_{fDn}}{R}_{2}$$

where

5 $${R}_{1}=\sum _{i=1}^{k-1}{\tilde{q}}_{fDni}(\frac{1}{2}+k-i){\rm{\Delta }}{z}_{Dn}^{2}+{\tilde{q}}_{fDnk}\frac{{\rm{\Delta }}{z}_{Dn}^{2}}{8}$$

and

6 $${R}_{2}={\tilde{q}}_{whfDn}({z}_{whfDn},s){z}_{fDnk}$$

Where ${\tilde{p}}_{fDnk}$ is the dimensionless pressure of Laplace domain at *k*th segment on the *n*th fracture; ${\tilde{p}}_{fDn}(0,s)$ is dimensionless average pressure of Laplace domain at the *n*th fracture; ${\tilde{q}}_{fDni}$ is the dimensionless fracture flux of Laplace domain at *i*th segment on the *n*th fracture; Δ*z*_*Dn*_ is the dimensionless length of fracture segment on the *n*th fracture; *Z*_*fDnk*_ is the dimensionless midpoint at *k*th segment on the *n*th fracture; *Z*_*whfDn*_ is the dimensionless well location on the *n*th fracture; *C*_*fDn*_ is the fracture conductivity on the *n*th fracture.

### Semi-analytical Solution

The reservoir model and fracture model are presented in details in Appendix B and C of Supplementary material. Now, we divide per fracture into *N*_*sn*_ segments to Eq. (1), thus, the *j*th pressure segment of *n*th fracture can be given by

7 $${\tilde{p}}_{fDj}=\sum _{n=1}^{{N}_{f}}\sum _{i=1}^{Nsn}\frac{{\tilde{q}}_{fDni}({u}_{Dn},s)}{{L}_{fDn}}{\int }_{(i-1){\rm{\Delta }}x}^{{\rm{\Delta }}x}{F}_{j}d{u}_{Dn}$$

where

8 $$\begin{array}{c}{F}_{j}({x}_{Dj},{y}_{Dj},{x}_{fDn},{y}_{fDn},{\theta }_{fn},s)\\ \,={K}_{0}[\sqrt{sg(s)}\sqrt{{({x}_{Dj}-{x}_{fDn}-{u}_{Dn}\cos {\theta }_{fn})}^{2}+{({y}_{Dj}-{y}_{fDn}-{u}_{Dn}\sin {\theta }_{fn})}^{2}}]\end{array}$$

The flow rate of each fracture should be equal to the sum of the flow flux of all fracture segment in this fracture, therefore, we have

9 $$\sum _{i=1}^{{N}_{sn}}{\tilde{q}}_{fDni}{\rm{\Delta }}{x}_{Dn}={\tilde{q}}_{whfDn}$$

The pressure should be equal for all fractures connected with horizontal wells, we have

10 $${\tilde{p}}_{fD1}(0,s)={\tilde{p}}_{fD2}(0,s)\ldots ={\tilde{p}}_{fD{N}_{f}-1}(0,s)={\tilde{p}}_{fD{N}_{f}}(0,s)$$

In addition, total rate of all fractures connected with horizontal wells should be equal to flow rate of horizontal wells, we have

11 $$\sum _{i=1}^{{N}_{f}}{\tilde{q}}_{whfDn}=\frac{1}{s}$$

Coupling the multi-branched fractures and reservoir-flow models at the fracture faces, we obtain a system of *N*_*ts*_ + *N*_*f*_ + *N*_*f*_ equations with *N*_*ts*_ + *N*_*f*_ + *N*_*f*_ unknowns. *N*_*ts*_ is the total number of fracture segments, and *N*_*f*_ is the total number of multi-branched fractures. Equations 7 to 11 constitute a system of linear equations that can be solved by Gauss elimination. Once the equations are solved, we can obtain the flux of each fracture segment. By substituting the flux of all fractures segment into equation 7, we can obtain bottom-hole pressure.

## Results

### Comparison with numerical simulation

**Figure 4:**
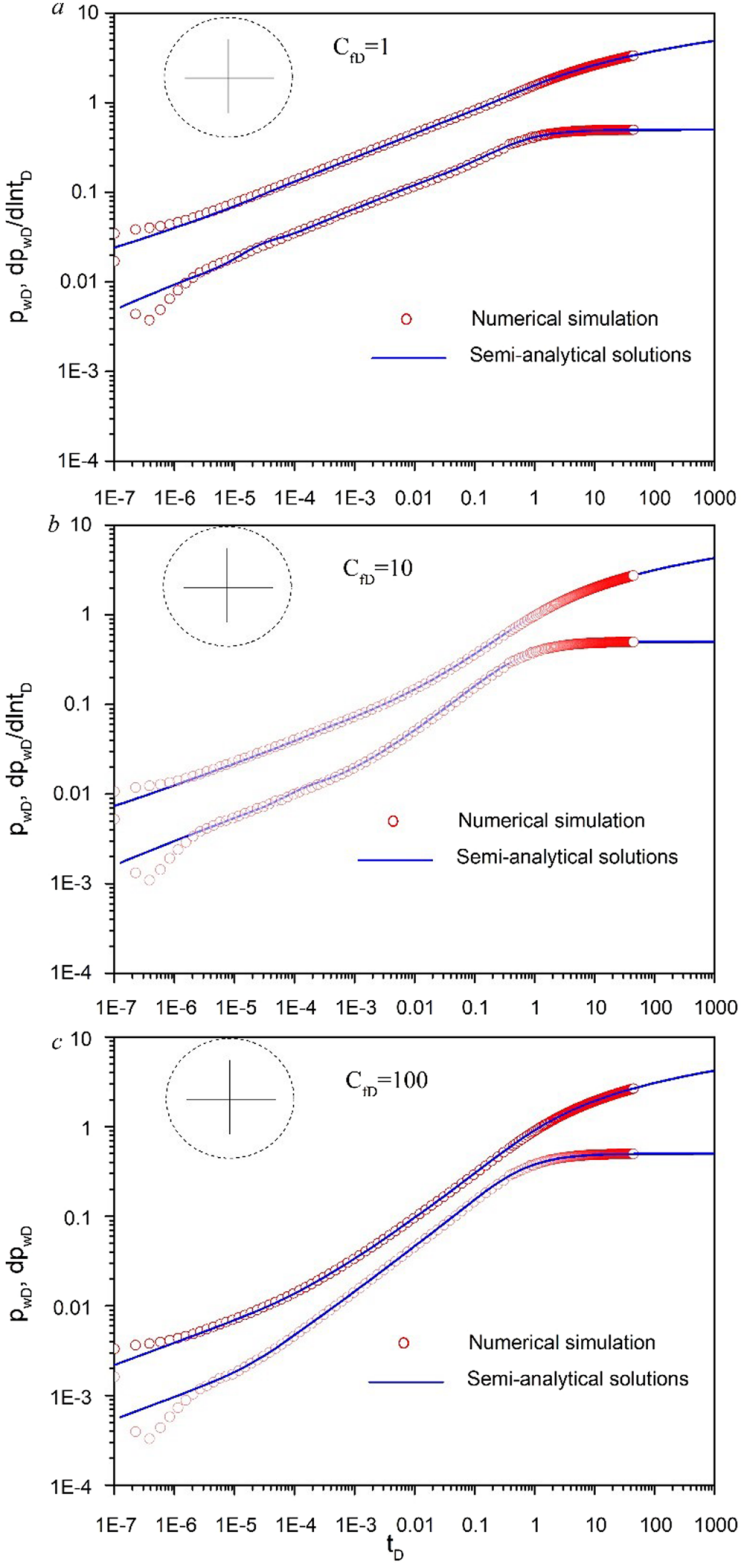
Validation for numerical simulation.

Figure 4 shows the comparison between results from this work and those computed data from commercial software Eclipse with four fractures. All the coordinates of the starting point for sources (*x*_*fD1*_, *y*_*fD1*_), (*x*_*fD2*_, *y*_*fD2*_), (*x*_*fD3*_, *y*_*fD3*_) and (*x*_*fD4*_, *y*_*fD4*_) are set to (3,3). Initial fracture conductivity *C*_*fD1*_, *C*_*fD2*_, *C*_*fD3*_ and *C*_*fD4*_ for all the fractures is set to 1, 10, 100 for three cases to compare with numerical simulation. Fracture length *L*_*fD1*_, *L*_*fD2*_, *L*_*fD3*_ and *L*_*fD4*_ for all the fractures is set to 0.5. The fracture angles *θ*_*f1*_, *θ*_*f2*_, *θ*_*f3*_ and *θ*_*f4*_ are set to π/2, π, 3π/2, 2π, respectively. The g(s) in Eq. (8) is set to be 1. The software is used to simulate the oil, gas and water flow during the reservoir development. The code is written using finite difference method. The fracture number is set to 4 and fracture conductivity *C*_*fD*_ is set to 1, 10, 100 in the software Eclipse. The comparison suggests that our model can be well validated by the software. However, for early time, the results from numerical simulation cannot well agree with the semi-analytical results and the accuracy of numerical simulation is controlled by the number of grids near the wellbore. Both pressure and pressure derivative data deviates from 1/4 slope straight line.

### Comparison with existing an analytical solution for a single fracture

**Figure 5:**
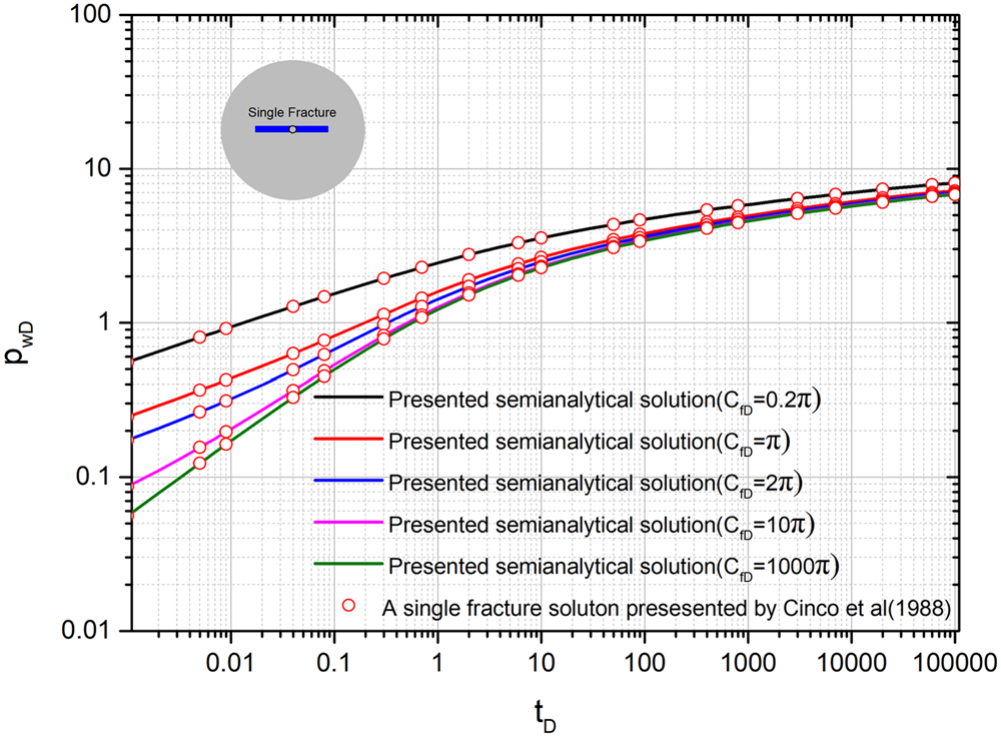
Comparison with existing analytical solution for a single fracture.

Figure 5 shows the comparison with existing analytical solutions presented by Cinco *et al*. (1988). The existing analytical solutions are special cases of our presented model. Therefore, the coordinates of the starting point for sources (*x*_*fD1*_, *y*_*fD1*_) and (*x*_*fD2*_, *y*_*fD2*_), are set to (3,3). Initial fracture conductivity *C*_*fD1*_ and *C*_*fD2*_ for all the fractures is set to 0.2π, π, 2π, 10π, and 1000π for five different cases. Fracture length *L*_*fD1*_ and *L*_*fD2*_ for both the fractures is set to 0.5. The fracture angles *θ*_*f1*_ and *θ*_*f2*_ are set to 0, π, respectively. The comparison shows that our model can be well validated by the solutions from literatures. However, in the literature, the solution is only considered into a single fracture as shown in Fig. 5. Therefore, our presented model is a general model, which could consider more complex branched fractures and inter-porosity flow effect.

### Comparison with numerical simulation for a single fracture with non-Darcy flow

**Figure 6:**
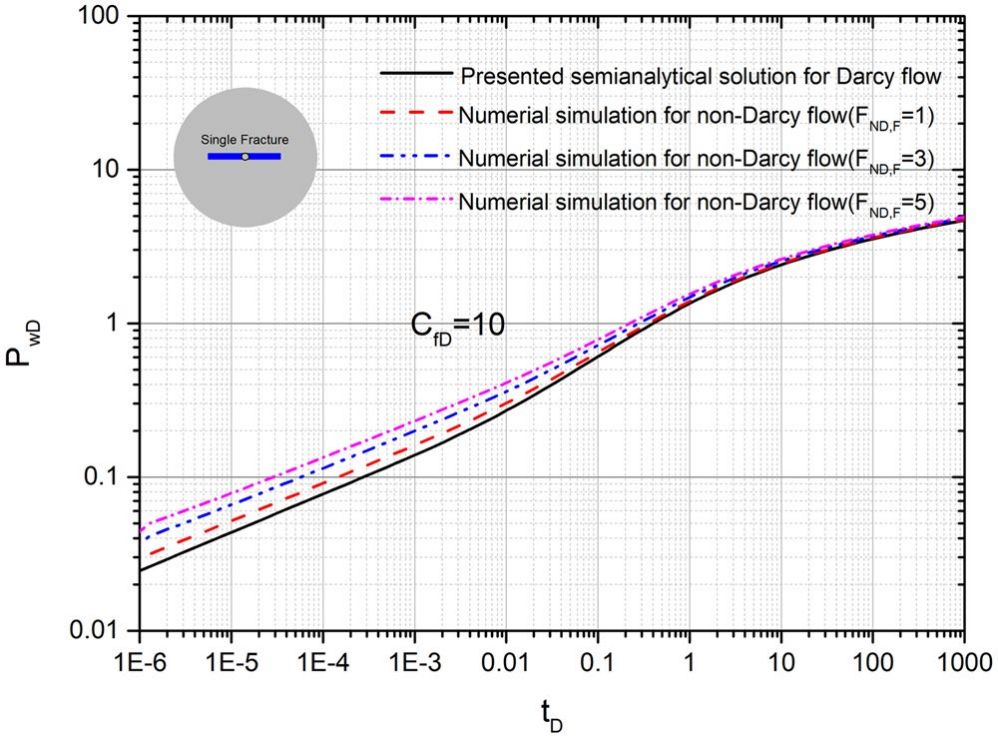
Comparison with numerical simulation for a single fracture with non-Darcy flow.

Figure 6 shows the graph of dimensionless pressure vs. dimensionless time for different values of non-Darcy Forchheimer number *F*_*ND,F*_ at the same fracture conductivity. We compared the presented semi-analytical solution with Darcy flow and numerical simulation with non-Darcy flow (different Forchheimer number values). The coordinates of the starting point for sources (*x*_*fD1*_, *y*_*fD1*_) and (*x*_*fD2*_, *y*_*fD2*_), are set to (3,3). Initial fracture conductivity *C*_*fD1*_ and *C*_*fD2*_ for all the fractures is set to 10. Fracture length *L*_*fD1*_ and *L*_*fD2*_ for both the fractures is set to 0.5. The fracture angles *θ*_*f1*_ and *θ*_*f2*_ are set to 0, π, respectively. The Forchheimer number values are set to 1, 3, 5, respectively. As expected, the Forchheimer number, *F*_*ND,F*_, has a observable effect on transient pressure behavior. In the early time when t_D_ < 1, A bigger Forchheimer number will lead to a stronger non-Darcy flow. We can see that in the early time the pressure becomes larger with the increase of Forchheimer number *F*_*ND,F*_, which implies non-Darcy flow will lead to a bigger pressure depletion. However, in the late time when t_D_ > 1, there is almost no error between Darcy flow and non Darcy flow.

### Flow Characteristics analysis

**Figure 7:**
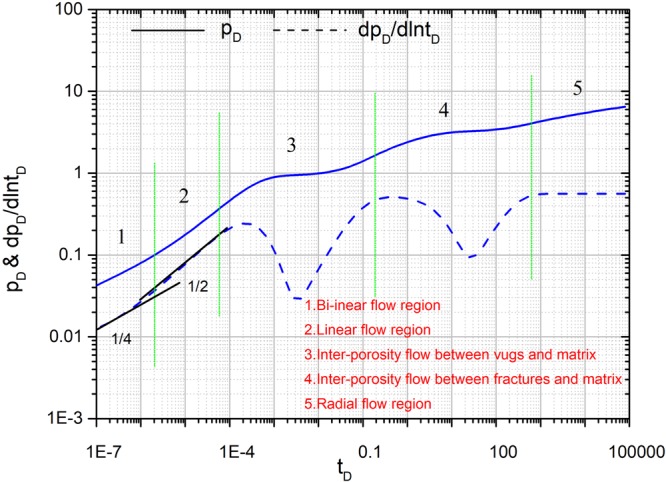
Flow characteristics analysis for multi-branched fractures in naturally fractured-vuggy reservoirs.

The transient flow characteristics are analyzed by type curves, which can be used to analyze transient pressure so as to recognize the fluids flow characteristics in fractured-vuggy reservoirs^21,22^. Fracture number is set to 4. All the coordinates of the starting point for sources (*x*_*fD1*_, *y*_*fD1*_), (*x*_*fD2*_, *y*_*fD2*_), (*x*_*fD3*_, *y*_*fD3*_) and (*x*_*fD4*_, *y*_*fD4*_) are set to (3,3). Initial fracture conductivity *C*_*fD1*_, *C*_*fD2*_, *C*_*fD3*_ and *C*_*fD4*_ for all the fractures is set to 2π for three cases to compare with numerical simulation. Fracture length *L*_*fD1*_, *L*_*fD2*_, *L*_*fD3*_ and *L*_*fD4*_ for all the fractures is set to 0.5. The fracture angles *θ*_*f1*_, *θ*_*f2*_, *θ*_*f3*_ and *θ*_*f4*_ are set to π/2, π, 3π/2, 2π, respectively. Fracture-vug, fracture-matrix, and vug-matrix inter-porosity flow coefficients are set to 3, 0.01 and 0.01, respectively. Vugs, fracture, matrix storage coefficients are set to 0.04, 0.005 and 0.955, respectively. Five main flow regions are well shown in Fig. 7.

1. A 1/4 slope straight line on the pressure derivative curve represents bi-linear flow region.
2. A 1/2 slope straight line on the pressure derivative curve represents linear flow region.
3. The first V-shaped segment on the pressure derivative curve represents inter-porosity flow region from vug system to the fracture system shows. After bi-linear flow and linear flow, the fluid in the fracture gradually decreases, so the fluid in the vugs will flow towards the fracture ^**46**^.
4. The second V-shaped segment on the pressure derivative curve represents inter-porosity flow region from matrix system to fracture system and vug system to matrix system. During this period, the fluid in the fracture is further reduced, and the fluid in the matrix also begins to flow towards the fracture. Because of the large amount of fluid stored in the vugs, the part of fluid in the vugs also begins to flow towards the fracture and other part of fluids in vugs flow towards the matrix^9^.
5. The radial flow regime shows a zero slope and 0.5 constant straight line on the pressure derivative curve. At the late time of the flow, the fluid far from the wellbore began to flow. At this time, the fluid around the wellbore converge toward the wellbore, which is a radial flow. During this period, fluid exchange in matrix, fracture and vugs is in dynamic balance.

### Parameter Influence

**Figure 8:**
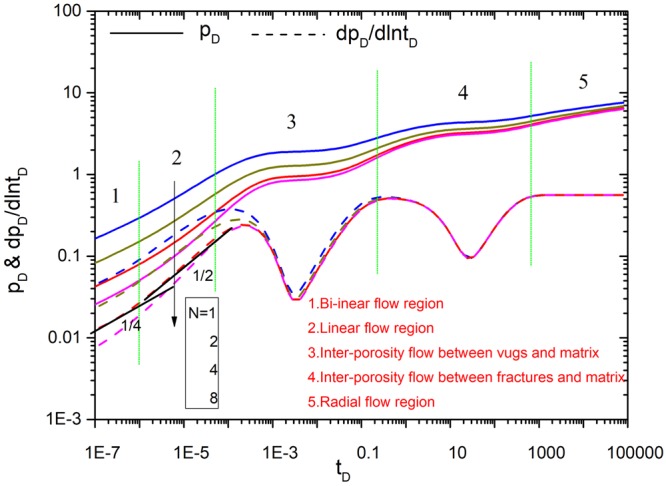
The effect of fracture number on type curves.

**Figure 9:**
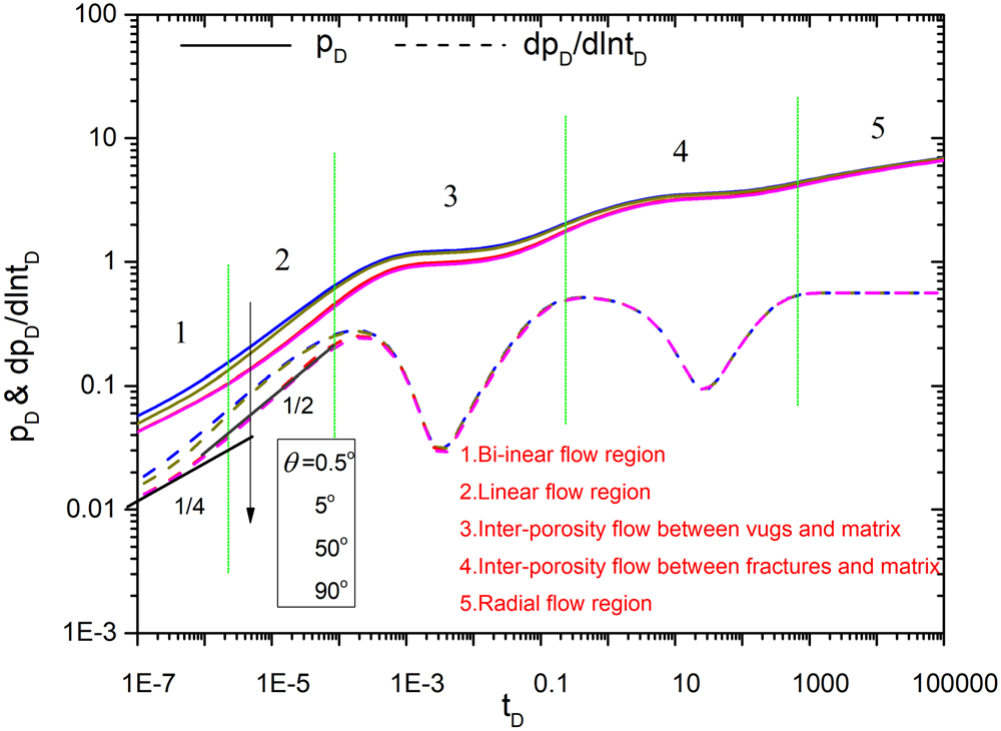
The effect of fracture angle on type curves.

**Figure 10:**
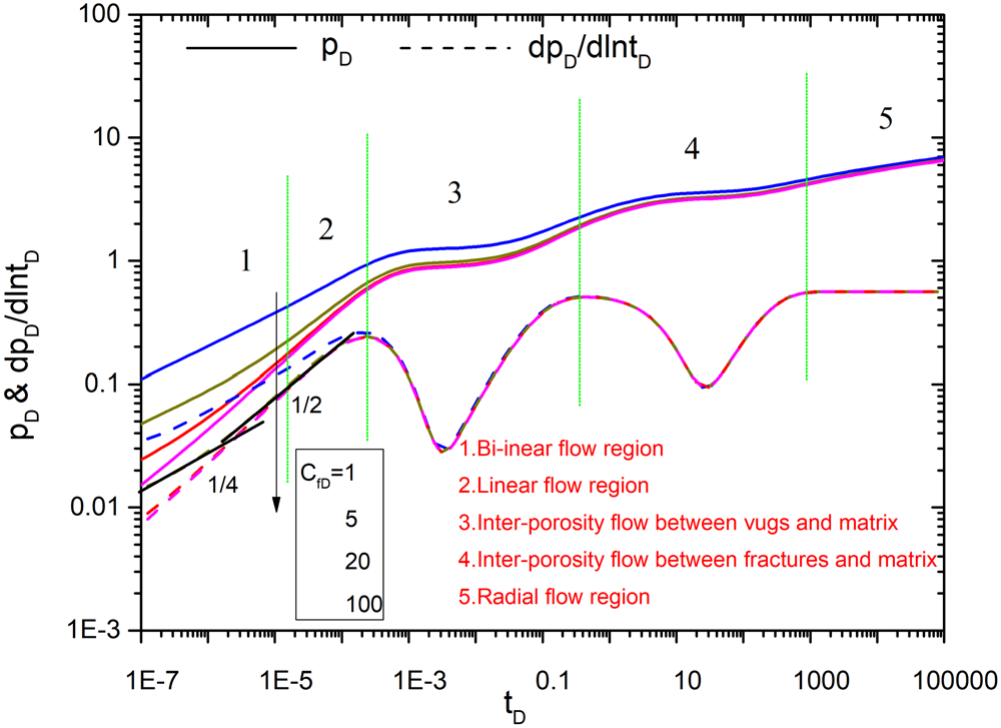
The effect of same fracture conductivity *C*_*fD*_ on type curves.

**Figure 11:**
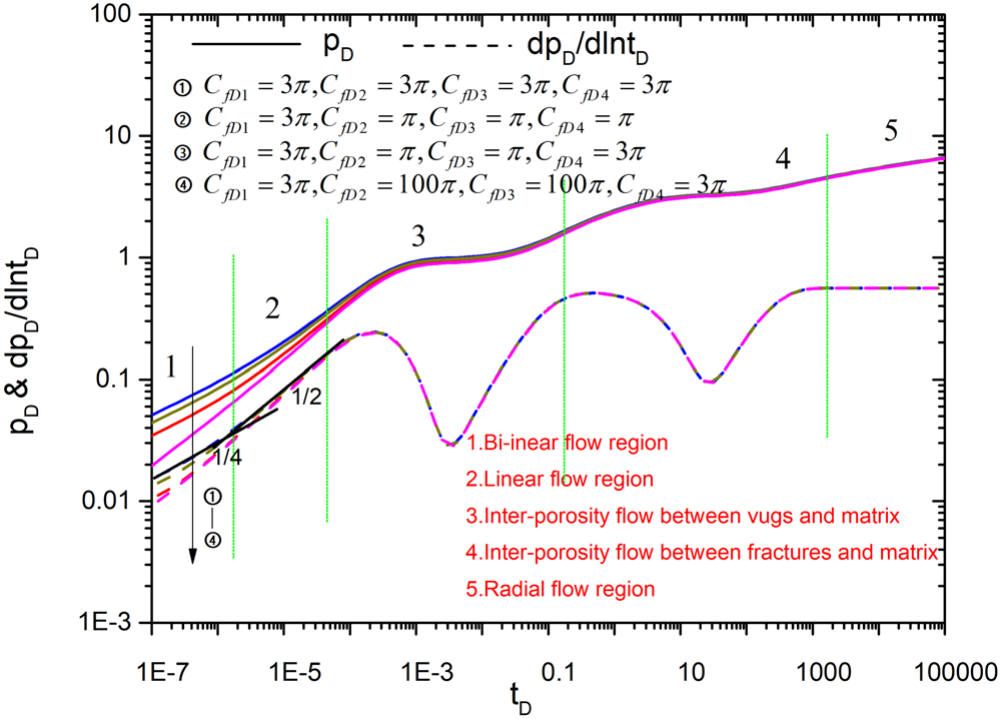
The effect of different fracture conductivity *C*_*fDn*_ on type curves.

**Figure 12:**
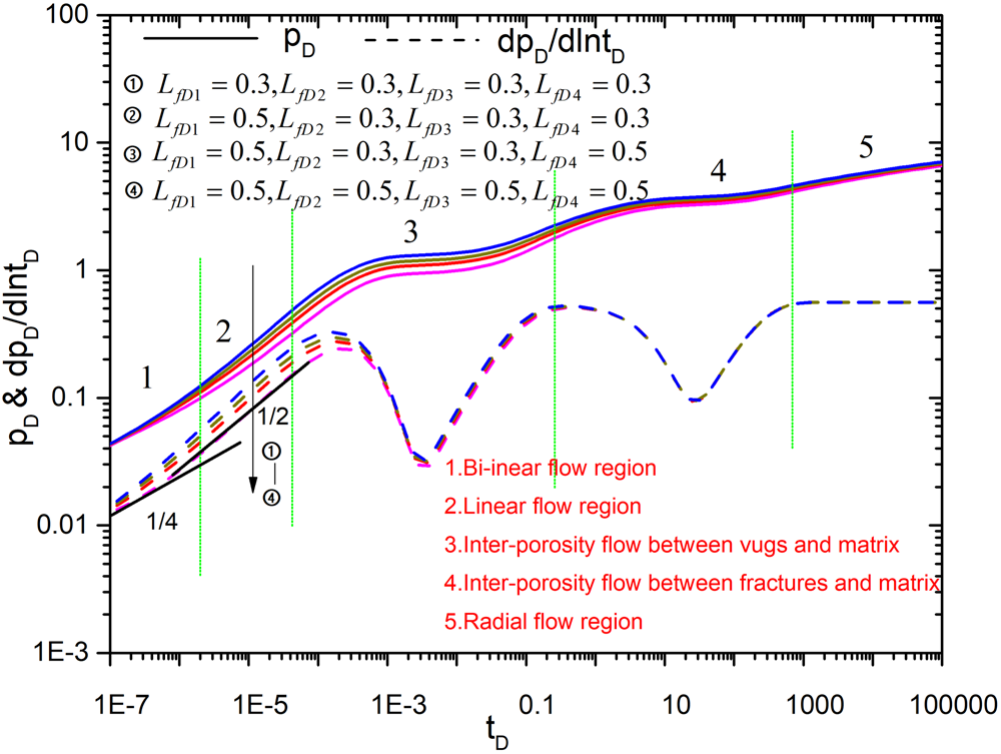
The effect of fracture length *L*_fDn_ on type curves.

Figures 8–12 show the influence of parameters (fracture number *N*, fracture angle *θ*, conductivity *C*_fD_, and fracture length *L*_fDn_) on pressure and pressure derivative curves. The initial parameters are listed here. The initial fracture number is set to 4. All the coordinates of the starting point for sources (*x*_*fD1*_, *y*_*fD1*_), (*x*_*fD2*_, *y*_*fD2*_), (*x*_*fD3*_, *y*_*fD3*_) and (*x*_*fD4*_, *y*_*fD4*_) are set to (3,3). Initial fracture conductivity *C*_*fD1*_, *C*_*fD2*_, *C*_*fD3*_ and *C*_*fD4*_ for all the fractures is set to 2π for three cases to compare with numerical simulation. Fracture length *L*_*fD1*_, *L*_*fD2*_, *L*_*fD3*_ and *L*_*fD4*_ for all the fractures is set to 0.5. The fracture angles *θ*_*f1*_, *θ*_*f2*_, *θ*_*f3*_ and *θ*_*f4*_ are set to π/2, π, 3π/2, 2π, respectively. Fracture-vug, fracture-matrix, and vug-matrix inter-porosity flow coefficients are set to 3, 0.01 and 0.01, respectively. Vugs, fracture, matrix storage coefficients are set to 0.04, 0.005 and 0.955, respectively.

As shown in Fig. 8, the dimensionless pressure decreases with the increase of the fracture number, which implies that it is the more the number of fractures, the smaller the pressure depletion. Therefore, pressure depletion could be reduced and well production can be improved through producing more fractures. The pressure derivative curves show that fractures number is mainly affecting flow behavior of bi-linear and linear flow region.

Figure 9 shows that facture angle affects the pressure responses and fracture number is 4. It is shown that the smaller the fracture angle is, the greater the pressure loss is. Pressure derivative curves show that when the fracture angle is greater than 1°, the bi-linear flow and the linear flow can be affected importantly by fracture angle. When the angle of the fractures is greater than 50°, the flow characteristic in early time is nearly not affected by the angle.

Figure 10 shows the effect of the conductivity on type curves. Both the pressure and its derivative curves show this effect mainly occurs in the early time. The pressure curve shows the dimensionless pressure increases as the conductivity decreases, which means a low conductivity will cause big pressure depletion. Pressure derivative curve shows bi-linear flow will end early as the conductivity increases. Linear flow cannot be observed when the conductivity is less than 1 and bi-linear flow cannot be observed when the conductivity is more than 20.

If the conductivity for each fracture is different, how the conductivity affects the type curves? As shown in Fig. 11, the uniform conductivity for case 3 exhibits the complete flow characteristics, the starting time of the linear flow becomes late and the pressure depletion increases as the each fracture conductivity decreases. This point is coincided with the analysis above under the uniform conductivity. Figure 12 shows the effect of the fracture length on type curves. The curves exhibit the complete flow characteristics when all fracture length is set to be 0.5. However, the pressure depletion will increase as fracture length is reduced. The effect of fracture length on flow behavior occurs in the early time. Bi-linear flow and linear flow characteristics are not observed when the fracture length is reduced. In summary, effect of fracture number on type curves is big and influence of other parameters on them is small.

The model proposed in this paper can well reveal the formation bilinear flow and the linear flow pattern and effects of some important parameters on bilinear flow and linear low are presented. In well test, because of the short test time, late data cannot be obtained. Therefore, the bilinear flow and the linear flow patterns are very important for early interpretation of production data. In addition, in this paper, we analyzed the influence of fracture correlation parameters on type curves. Because the fracture related parameters are all affecting flow near wellbore, we can see that the parameters only affect bilinear flow and linear flow. However, the region 4 and region 5 can be affected by inter-porosity flow coefficient and storage coefficient, which have been analyzed in previous literatures^9,34^. However, flow pattern in early time is not revealed and production data in early time cannot be analyzed in previous literatures. We presented a comprehensive model considering all possible flow patterns in this paper and that we can analyze the production data of bilinear flow and linear flow is the important contribution of this paper.

## Discussions

### Advantages of the presented semi-analytical model

The semi-analytical method effectively eliminates the truncation error and has the same order of accuracy as the analytical method if the property varication is not considered. The semi-analytical method does not require discretization of reservoir at the first step of simulation and it also it does not need discretization of time. It can be done at any time. The disadvantage is that at present our semi-analytical model is now limited to single-phase flows. However, in the future, we are trying to study the semi-analytical solution of two-phase flow.

### Application of a real field case

What is the most striking is that this semi-analytical model can be applied to obtain more parameters reflecting comprehensive flow characteristics through using history matching procedure. A carbonate reservoir can be characterized by many parameters, i.e. fracture conductivity, fracture number, fracture angle, fracture length. If those parameters are unknown, the determination of unknown parameters is in fact a subject of inverse problems. Determining those unknown parameters could use the present solution combining with the algorithm of auto history matching^45^.

**Figure 13:**
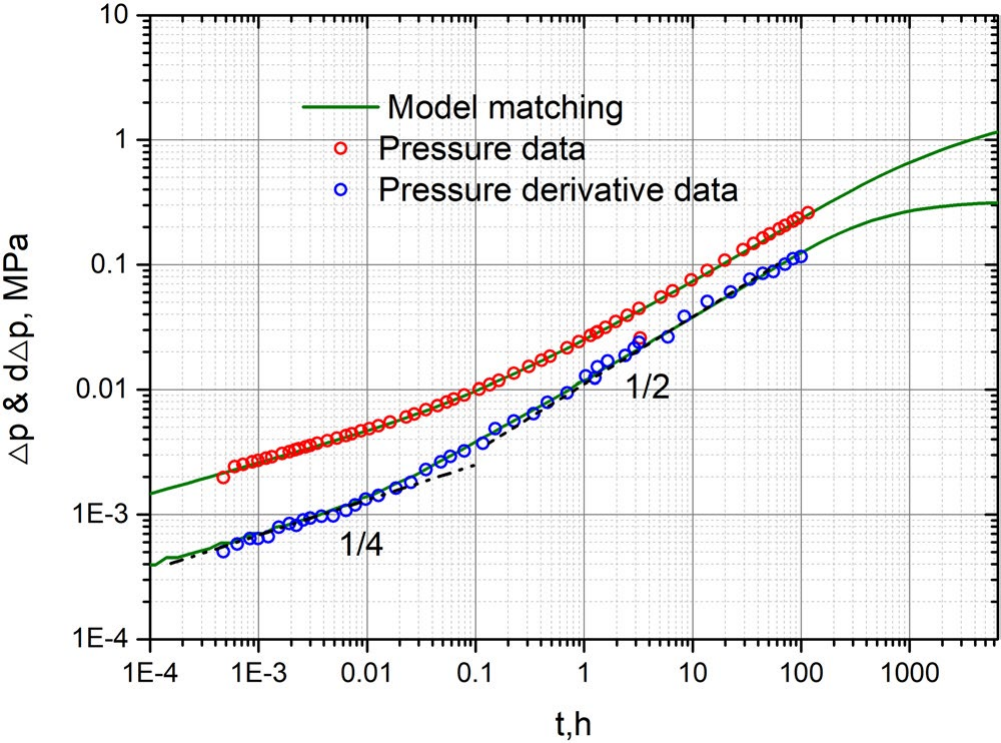
Matching of well test data using the presented semi-analytical model.

In this section, a field case is selected to validate the presented solution. Well A located in the western oil field of China is selected. The well test data is shown in Fig. 13. The reservoir thickness is 25 m and the effective porosity (matrix + fractures + vugs) is 0.12. The oil viscosity is 0.00152 Pa▪s and the total compressibility is 0.0014 MPa^−1^. The production of well is 120 m3/d. According to well test data, we have drawn the pressure and pressure derivative curves in Fig. 13. Pressure derivative curves show 1/4 and 1/2 slopes straight lines, which implies that bi-linear flow and linear flow occur in the early time. However, the inter-porosity flow region is not found in the figure. Therefore, g(s) is set to 1 in our presented model. Furthermore, we can determine those unknown parameters use the present solution combining with the algorithm of auto history matching^45^. The final matching results are given: The permeability k_f_ is 23.2Md; fracture half length is 430 m; fracture number is 4; fracture conductivity is 99.7. From fracture conductivity, we can see that fracturing effects are good. The matching results also implies that the presented model can be used to explain the well test data in the early time, which cannot be done in previous study^34^.

## Conclusions

Based on our work, the following conclusions can be drawn.

1. In this paper, firstly a semi-analytical solution for finite conductivity multi-branched fractures is presented.
2. The presented semi-analytical solutions are validated by numerical simulation and existing analytical solutions.
3. The flow Characteristics are also proposed in this article, and flow in carbonate reservoirs can be divided into five regions, e.g. bi-linear flow region, linear flow region, inter-porosity flow region between vugs and fractures, inter-porosity region between matrix and fractures, and radial flow region.
4. The influence factors analysis shows fracture number will be the main factor influencing on flow. The present solution combining with the algorithm of auto history matching to obtain reservoir parameters, furthermore, production performance analysis can be done.

## Electronic supplementary material

Appendix
