# Supplementary material for: A Novel Semi-Analytical Model for Multi-branched Fractures in Naturally Fractured-Vuggy Reservoirs
Source: Sci Rep. 2018 Aug 2;8:11586. doi: 10.1038/s41598-018-30097-2 (PMC6072746; doi:10.1038/s41598-018-30097-2)
Supplement: Supplementary file 1 — Appendix [file 41598_2018_30097_MOESM1_ESM.doc]

**SUPPLEMENTAL MATERIALS**

**A Novel Semi-Analytical Model for Multi-branched Fractures in Naturally Fractured-Vuggy Reservoirs**

**Wang Leia[[1]](#footnote-2) *, Xiaoxia Chen^b^, Zunyi Xia^a^**

**Author Affiliations:** ^a^ BIC-ESAT, College of Engineering, Peking University, Beijing, China, 100871**.** ^b^ China University of Geosciences, Beijing, China,100083

**Corresponding Authors:**

**Lei Wang**, BIC-ESAT, College of Engineering, Peking University, Beijing, China, 100083**.**  Phone Number: +86 13021906144; Fax Number: +8610-62751812.

E-mail:wanglei1986sp@foxmail.com.

**Running Title:**

**The Appendix** of the main article file named as "A Novel Semi-Analytical Model for Multi-branched Fractures in Naturally Fractured-Vuggy Reservoirs".

**Conflict of Interest:**

We declare that we have no competing interests.

**Appendix A: Nomenclature and dimensionless definitions of variables**

The dimensionless pressure

$p_{jD}=\frac{2\pi k_{f}h(p_{i}-p_{j}(r,t))}{\mu Q} j\text{=f,m,v}$ (A-1)

Where: *p_fD_* is dimensionless pressure of fracture; *p_mD_* is dimensionless pressure of matrix; *p_vD_* is dimensionless pressure of vugs; *p* and *p_i_* are respectively pressure and initial formation pressure, Pa. *k_f_* is the fracture permeability, m^2^; *h* is formation thickness, m; *µ* is fluid viscosity, Pa ▪s; *Q* is flow rate of a well in the wellhore , m3 /s;

The dimensionless time

(A-2)

Where: *t* is time variable, s; *t_D_* is dimensionless time; *L* is the reference length, m; φ_m_ is matrix porosity; φ_v_ is vugs porosity; φ_f_ is fracture porosity; cm is the matrix compressibility, 1/Pa; *c_v_* is the vugs compressibility, 1/Pa; *c_f_* is the fracture compressibility, 1/Pa.

Fracture storage coefficient

(A-3)

Vug storage coefficient

(A-4)

Matrix storage coefficient

(A-5)

Fracture-Matrix inter-porosity flow coefficient

$\lambda_{fm}=\frac{\alpha_{fm}L^{2}k_{m}}{k_{f}}$ (A-6)

Vug-Matrix inter-porosity flow coefficient

$\lambda_{vm}=\frac{\alpha_{vm}L^{2}k_{m}}{k_{f}}$ (A-7)

Fracture-Vug inter-porosity flow coefficient

$\lambda_{fv}=\frac{\alpha_{fv}L^{2}k_{v}}{k_{f}}$ (A-8)

Where: *k_f_* is the fracture permeability, m^2^; *k_v_* is the vug permeability, m^2^; *k_m_* is the matrix permeability, m^2^.

*α_fv_*, *α_vm_*, *α_fm_* are the inter-porosity flow shape factors. The shape factors for f-m or v-m is defined by Warren and Root (1963). Kazemi et al. (1976) proposed the following first approximation for the shape factor of rectangular matrix blocks of dimension *L_x_*×*L_y_*×*L_z_*:

$\alpha_{fm}=\alpha_{vm}=\alpha=4\left(\frac{1}{L_{x}^{2}}+\frac{1}{L_{y}^{2}}+\frac{1}{L_{z}^{2}}\right)$ (A-9)

For the purposes of this paper, we will assume square matrix blocks of size *L_R_* and use *α*=12/*L_R_* to compute the shape factor. For f-v interaction, the shape factor for vugs is defined as

$\alpha_{fv}=\frac{A_{fv}}{l_{fv}}$ (A-10)

Where: *A_fv_* is the total fracture and vug connection area per unit volume of rock (m^2^ /m^3^) and *l_fv_* is characteristic length, defined as:

$l_{fv}=\frac{l_{f}}{2}$ (A-11)

where *l_f_* is the average length of small fractures that connect vugs with fractures.

Dimensionless fracture flow rate

$q_{fDn}=\frac{L_{fn}q_{fn}}{wQ}$ (A-12)

Dimensionless fracture position

$L_{fDn}=\frac{L_{fn}}{L}$ $x_{fDn}=\frac{x_{fn}}{L}$ $y_{fDn}=\frac{y_{fn}}{L}$ $x_{D}=\frac{x}{L}$ $y_{D}=\frac{y}{L}$ (A-13)

Where: *L_fn_* is the half length of *n*th fracture, m; *w* is the width of fracture, m;.*q_fn_* is flow rate of *n*th fracture, m3/s; *L_fDn_* is dimensionless half length of *n*th fracture; (*x_fDn_*, *y_fDn_*) is the dimensionless endpoint coordinates of *n*th fracture; (*x_fn_*, *y_fn_*) is the starting point of source coordinates of *n*th fracture, m; (*x_D_*, *y_D_*) is the dimensionless coordinates of any position; (*x*, *y*) is the dimensionless coordinates of any position, m.

**Appendix B: Derivation of details for reservoir model**

The first term and the second term in the left of Eq. (B-1) represent the difference between mass flow in and mass of flow out in the unit volume. The third term represents the source term in the multi-branched fractures. The fourth term in the left of governing equation represents inter-porosity flow volume from the matrix into the fractures. The fifth term in the left of governing equation represents inter-porosity flow volume from the vugs into the fractures. The first term in the right of the governing equation represents total mass change in the natural fracture. According to the assumptions above given, multi-branched fractures model for carbonate reservoirs could be established by using mass conversation

(B-1)

where

$S_{f}(t)=\sum_{n=1}^{N_{f}} \int_{0}^{L_{fn}} \frac{q_{fn}(u_{n},t)}{wh}\delta(x-x_{fn}-u_{n}\cos\theta_{fn})\delta(y-y_{fn}-u_{n}\sin\theta_{fn})du_{n}$ (B-2)

Where, $\delta(x)$is the dirac function; $x_{fn}$and $y_{fn}$ are the coordinates of the starting point for sources of nth fracture, m; $\theta_{fn}$ is the angle between the *n*th fracture and *x* axis; $u_{n}$is point source position of *n*th fracture, integral variable; and other parameters are defined in **Appendix A**.

When vugs are regarded as unit volume, the total rate change in unit volume of the vugs is the sum of inter-porosity outflow volume from vugs into the fractures, and inter-porosity outflow volume from the vugs into the matrix,

(B-3)

Similarly, when matrix are regarded as the unit volume, the total rate change in unit volume of the matrix is the sum of inter-porosity outflow volume from matrix into the fractures, and inter-porosity inflow volume from the vugs into the matrix.

(B-4)

The initial condition is

$p_{f}(r,0)=p_{v}(r,0)=p_{m}(r,0)=p_{i}$ (B-5)

Where, *p_i_* is the initial pressure, Pa. Outer boundary at constant pressure for infinite system can be expressed as

$p_{f}(r\to\infty,t)=p_{v}(r\to\infty,t)=p_{m}(r\to\infty,t)=p_{i}$ (B-6)

Eqs. (B-1)-(B-6) can be redefined by using dimensionless quantities listed in **Appendix A**. The dimensionless equations are defined as following

$\frac{\partial^{2}p_{fD}}{\partial x_{D}^{2}}+\frac{\partial^{2}p_{fD}}{\partial y_{D}^{2}}+S_{fD}(t_{D})+\lambda_{fv}(p_{vD}-p_{fD})+\lambda_{fm}(p_{mD}-p_{fD})=w_{f}\frac{\partial p_{fD}}{\partial t_{D}}$ (B-7)

Where, all dimensionless definitions are shown in **Appendix A**,

$S_{fD}(t_{D})=\sum_{n=1}^{N_{f}} \int_{0}^{L_{fDn}} \left[\begin{matrix} \frac{2\pi}{L_{fDn}}q_{fDn}(u_{Dn},t_{D})\delta(x_{D}-x_{fDn}-u_{Dn}\cos\theta_{fDn}) \\ \times\delta(y_{D}-y_{fDn}-u_{Dn}\sin\theta_{fDn}) \end{matrix}\right]du_{Dn}$ (B-8)

$\omega_{v}\frac{\partial p_{vD}}{\partial t_{D}}+\lambda_{fv}(p_{vD}-p_{fD})+\lambda_{vm}(p_{vD}-p_{mD})=0$ (B-9)

$\omega_{m}\frac{\partial p_{mD}}{\partial t_{D}}+\lambda_{fm}(p_{mD}-p_{fD})-\lambda_{vm}(p_{vD}-p_{mD})=0$ (B-10)

Initial condition becomes

$p_{fD}(x_{D},y_{D},0)=p_{vD}(x_{D},y_{D},0)=p_{mD}(x_{D},y_{D},0)=0$ (B-11)

Outer boundary condition becomes

$p_{fD}(\pm\infty,y_{D},t_{D})=0$ (B-12)

and

$p_{fD}(x_{D},\pm\infty,t_{D})=0$ (B-13)

The Laplace transform is based on $t_{D}$ and functions as follows

$\tilde{p}_{D}(r_{D},s)=\int_{0}^{\infty} p_{D}(r_{D},t_{D})e^{-st_{D}}dt_{D}$ (B-14)

Applying the Laplace transformation to Eqs.(6)-(10), we have

$\frac{\partial^{2}\tilde{p}_{fD}}{\partial x_{D}^{2}}+\frac{\partial^{2}\tilde{p}_{fD}}{\partial y_{D}^{2}}+\tilde{S}_{fD}(s)+\lambda_{fv}(\tilde{p}_{vD}-\tilde{p}_{fD})+\lambda_{fm}(\tilde{p}_{mD}-\tilde{p}_{fD})=sw_{f}\tilde{p}_{fD}$ (B-15)

$\tilde{S}_{fD}(s)=\sum_{n=1}^{N_{f}} \int_{0}^{L_{fDn}} \left[\begin{matrix} \frac{2\pi}{L_{fDn}}\tilde{q}_{fDn}(u_{Dn},s)\delta(x_{D}-x_{fDn}-u_{Dn}\cos\theta_{fDn}) \\ \times\delta(y_{D}-y_{fDn}-u_{Dn}\sin\theta_{fDn}) \end{matrix}\right]du_{Dn}$ (B-16)

$\omega_{v}s\tilde{p}_{vD}+\lambda_{fv}(\tilde{p}_{vD}-\tilde{p}_{fD})+\lambda_{vm}(\tilde{p}_{vD}-\tilde{p}_{mD})=0$ (B-17)

$\omega_{m}s\tilde{p}_{mD}+\lambda_{fm}(\tilde{p}_{mD}-\tilde{p}_{fD})-\lambda_{vm}(\tilde{p}_{vD}-\tilde{p}_{mD})=0$ (B-18)

Substituting the vug and matrix equations of (B-17) and (B-18) into the fracture equation (B-15), we can obtain

$\frac{\partial^{2}\tilde{p}_{fD}}{\partial x_{D}^{2}}+\frac{\partial^{2}\tilde{p}_{fD}}{\partial y_{D}^{2}}+\tilde{S}_{fD}(s)=sg(s)\tilde{p}_{fD}$ (B-19)

Where

$g(s)=w_{f}+\frac{(\lambda_{fv}+\lambda_{fm})s+\frac{1-\omega_{f}}{\omega_{v}\omega_{m}}\left[\lambda_{fv}\lambda_{fm}+(\lambda_{fv}+\lambda_{fm})\lambda_{vm}\right]}{s^{2}+\left[\frac{\lambda_{fv}}{\omega_{v}}+\frac{\lambda_{fm}}{\omega_{m}}+\left(\frac{1}{\omega_{v}}+\frac{1}{\omega_{m}}\right)\lambda_{vm}\right]s+\frac{\lambda_{fv}\lambda_{fm}+(\lambda_{fv}+\lambda_{fm})\lambda_{vm}}{\omega_{v}\omega_{m}}}$ (B-20)

Outer boundary condition in Laplace domain is

$\tilde{p}_{fD}(\pm\infty,y_{D},s)=0$ (B-21)

and

$\tilde{p}_{fD}(x_{D},\pm\infty,s)=0$ (B-22)

Through using double Fourier transform to Eqs.(B-19) and (B-22) (See **Appendix D**), it is easy to obtain the solution as shown in Eq.(B-23)

$\tilde{p}_{fD}(x_{D},y_{D},s)=\sum_{n=1}^{N_{f}} \int_{0}^{L_{fDn}} \frac{1}{L_{fDn}}\tilde{q}_{fDn}(u_{Dn},s)F(x_{D},y_{D},x_{fDn},y_{fDn},\theta_{fn},s)du_{Dn}$ (B-23)

Where

$F(x_{D},y_{D},x_{fDn},y_{fDn},\theta_{fn},s)=K_{0}\left[\sqrt{sg(s)}\sqrt{(x_{D}-x_{fDn}-u_{Dn}\cos\theta_{fn})^{2}+(y_{D}-y_{fDn}-u_{Dn}\sin\theta_{fn})^{2}}\right]$(B-24)

*K_0_*(*x*) is the modified Bessel function (2nd kind, 0 order).

**Appendix C: Derivation of details for fracture model**

According to mass balance condition, the fluid flow inside *n*th fracture in the system may be defined by the following equations

$\frac{\partial\left(\frac{k_{fn}}{\mu}\frac{\partial p_{fn}}{\partial z_{n}}\right)}{\partial z_{n}}+\frac{q_{whfn}}{w_{fn}h}\delta(z_{n})-\Omega_{fn}(z_{n},t)=0$ (C-1)

where

$\Omega_{fn}(z_{n},t)=\int_{0}^{L_{fn}} \frac{q_{zfn}(z_{n},t)}{w_{fn}hL_{fn}}\delta(z_{n}-\gamma_{n})d\gamma_{n}$ (C-2)

Where *p_fn_* the pressure in the *n*th fracture, Pa; *z_whfn_* is the *n*th well position in the fracture, m; *k_fn_* is the fracture permeability, m^2^; *q_whfn_* is *n*th well rate in the fracture, m^3^/s; *L_fn_* is the half length of *n*th, m; *γ_n_* is the source position of *n*th fracture; *h* is the reservoir thick, m; *q_zfn_* is the *n*th fracture flux, m^3^/s; *L* is the reference length, m; *z*_n_ is the coordinate along the fracture, m; *w_fn_* is the *n*th fracture width, m; *Q* is the reference rate, m^3^/s; *B* is volume coefficient. In order to simplify Eq.(19), we take the following dimensionless transforms:

$$P_{fD}=\frac{2\pi kh(p_{i}-p_{fn})}{BQ\mu}$$

$$C_{fDn}=\frac{k_{fn}w_{fn}}{kL_{fn}}$$

$$q_{whfDn}=\frac{q_{whfn}}{Q}$$

$$L_{fDn}=\frac{L_{fn}}{L}$$

$$\gamma_{Dn}=\frac{\gamma_{n}}{L}$$

$$z_{Dn}=\frac{z_{n}}{L}$$

$$q_{zfDn}=\frac{q_{zfn}}{Q}$$

Therefore, the following equations can be obtained

$\frac{\partial^{2}p_{fDn}}{\partial z_{Dn}^{2}}+\frac{2\pi q_{whfDn}}{L_{fDn}C_{fDn}}\delta(z_{Dn})-\int_{0}^{L_{fDn}} \frac{2\pi q_{zfDn}(z_{Dn},t_{D})}{L_{fDn}^{2}C_{fDn}}\delta(z_{Dn}-\gamma_{Dn})d\gamma_{Dn}=0$ (C-3)

The initial condition can be given by

$p_{fDn}(z_{Dn},0)=0$ (C-4)

The boundary conditions are given by

$\frac{\partial p_{fDn}(0,t_{D})}{\partial z_{Dn}}=0$ (C-5)

and

$\frac{\partial p_{fDn}(L_{fDn},t_{D})}{\partial z_{Dn}}=0$ (C-6)

Imposing Laplace transforms on *t_D_* in Eqs.(C-3)-(C-6), we have

$\frac{\partial^{2}\tilde{p}_{fDn}}{\partial z_{Dn}^{2}}+\frac{2\pi\tilde{q}_{whfDn}}{L_{fDn}C_{fDn}}\delta(z_{Dn})-\int_{0}^{L_{fDn}} \frac{2\pi\tilde{q}_{zfDn}(z_{Dn},s)}{L_{fDn}^{2}C_{fDn}}\delta(z_{Dn}-\gamma_{Dn})d\gamma_{Dn}=0$ (C-7)

The boundary conditions become

$\frac{\partial\tilde{p}_{fDn}(0,s)}{\partial z_{Dn}}=0$ (C-8)

and

$\frac{\partial\tilde{p}_{fDn}(L_{fDn},s)}{\partial z_{Dn}}=0$ (C-9)

The pressure-drop evaluation for a fracture is presented in the following equation. The pressure drop at *k*^th^ segment on the *n*^th^ fracture is given by

$\tilde{p}_{fDnk}(z_{Dnk},s)=\tilde{p}_{fDn}(0,s)+\frac{2\pi}{L_{fDn}^{2}C_{fDn}}R_{1}-\frac{2\pi}{L_{fDn}C_{fDn}}R_{2}$ (C-10)

where

$R_{1}=\sum_{i=1}^{k-1} \tilde{q}_{fDni}\left(\frac{1}{2}+k-i\right)\Delta z_{Dn}^{2}+\tilde{q}_{fDnk}\frac{\Delta z_{Dn}^{2}}{8}$ (C-11)

and

$R_{2}=\tilde{q}_{whfDn}(z_{whfDn},s)z_{fDnk}$ (C-12)

*H* is the Heaviside unit step function

Where $\tilde{p}_{fDnk}$ is the dimensionless pressure of Laplace domain at *k*th segment on the *n*th fracture; $\tilde{p}_{fDn}(0,s)$ is dimensionless average pressure of Laplace domain at the *n*th fracture; $\tilde{q}_{fDni}$ is the dimensionless fracture flux of Laplace domain at *i*th segment on the *n*th fracture; $\Delta z_{Dn}$ is the dimensionless length of fracture segment on the *n*th fracture; $z_{fDnk}$ is the dimensionless midpoint at *k*th segment on the *n*th fracture; $z_{whfDn}$ is the dimensionless well location on the *n*th fracture; $C_{fDn}$ is the fracture conductivity on the *n*th fracture.

**Appendix D: Fourier transform to reservoir model**

The partial differential equations of free space are as follows

$\frac{\partial^{2}\tilde{p}_{fD}}{\partial x_{D}^{2}}+\frac{\partial^{2}\tilde{p}_{fD}}{\partial y_{D}^{2}}+\tilde{S}_{fD}(s)=sg(s)\tilde{p}_{fD}$ (D-1)

$\tilde{S}_{fD}(s)=\sum_{n=1}^{N_{f}} \int_{0}^{L_{fDn}} \left[\begin{matrix} \frac{2\pi}{L_{fDn}}\tilde{q}_{fDn}(u_{Dn},s)\delta(x_{D}-x_{fDn}-u_{Dn}\cos\theta_{fDn}) \\ \times\delta(y_{D}-y_{fDn}-u_{Dn}\sin\theta_{fDn}) \end{matrix}\right]du_{Dn}$ (D-2)

Outer boundary condition in Laplace domain is

$\tilde{p}_{fD}(\pm\infty,y_{D},s)=0$ (D-3)

and

$\tilde{p}_{fD}(x_{D},\pm\infty,s)=0$ (D-4)

The Fourier transform on the variable *x*_D_ is defined as follows

$\bar{\tilde{p}}_{fD}(k)=\int_{-\infty}^{\infty} \tilde{p}_{fD}(x_{D})e^{-ikx_{D}}dx_{D}$ (D-5)

Inverse transformation formula is defined as

$\tilde{p}_{fD}(x_{D})=\frac{1}{2\pi}\int_{-\infty}^{\infty} \bar{\tilde{p}}_{fD}(k)e^{ikx_{D}}dk$ (D-6)

Through Fourier transform to *x*_D_ of Eq.(D-1) and Eq.(D-2), we obtain

$-k^{2}\bar{\tilde{p}}_{fD}+\frac{\partial^{2}\bar{\tilde{p}}_{fD}}{\partial y_{D}^{2}}-sg(s)\bar{\tilde{p}}_{fD}=-\bar{\tilde{S}}_{fD}(s)$ (D-7)

Eq.(A-2) becomes

$\bar{\tilde{S}}_{fD}(k,y_{D})=\sum_{n=1}^{N_{f}} \int_{0}^{L_{fDn}} \frac{2\pi}{L_{fDn}}\tilde{q}_{fDn}(u_{n},s)e^{-ik(x_{fDn}+u_{Dn}\cos\theta_{fn})}\delta(y_{D}-y_{fDn}-u_{Dn}\sin\theta_{fn})du_{Dn}$ (D-8)

The Fourier transform on the variable *y*_D_ is defined as follows

$\bar{\bar{\tilde{p}}}_{fD}(l)=\int_{-\infty}^{\infty} \bar{\tilde{p}}_{fD}(y_{D})e^{-ily_{D}}dy_{D}$ (D-9)

Through Fourier transform to *y*_D_ of Eq.(D-7) and Eq.(D-8), we obtain

$-k^{2}\bar{\bar{\tilde{p}}}_{fD}-l^{2}\bar{\bar{\tilde{p}}}_{fD}-sg(s)\bar{\bar{\tilde{p}}}_{fD}=-\bar{\bar{\tilde{S}}}_{fD}(s)$ (D-10)

Eq.(D-8) becomes

(D-11)

In summary, we have

$\bar{\bar{\tilde{p}}}_{fD}=\frac{\bar{\bar{\tilde{S}}}_{fD}(k,l)}{k^{2}+l^{2}+sg(s)}$ (D-12)

Imposing inverse Fourier transform to Eq.(D-12), we have

$\tilde{p}_{fD}(x_{D},y_{D})=\frac{1}{4\pi^{2}}\int_{-\infty}^{\infty} \int_{-\infty}^{\infty} \frac{\left[\sum_{n=1}^{N_{f}} \int_{0}^{L_{fDn}} \frac{2\pi}{L_{fDn}}\tilde{q}_{fDn}(u_{n},s)e^{-ik(x_{fDn}+u_{Dn}\cos\theta_{fn})}e^{-il(y_{fDn}+u_{Dn}\sin\theta_{fn})}du_{Dn}\right]}{k^{2}+l^{2}+s}e^{ily_{D}}e^{ikx_{D}}dldk$(D-13)

Through using the infinite integral formula, final solution can be given as

$\tilde{p}_{fD}(x_{D},y_{D},s)=\sum_{n=1}^{N_{f}} \int_{0}^{L_{fDn}} \frac{1}{L_{fDn}}\tilde{q}_{fDn}(u_{Dn},s)F(x_{D},y_{D},x_{fDn},y_{fDn},\theta_{fn},s)du_{Dn}$ (D-14)

$F(x_{D},y_{D},x_{fDn},y_{fDn},\theta_{fn},s)=K_{0}\left[\sqrt{sg(s)}\sqrt{(x_{D}-x_{fDn}-u_{Dn}\cos\theta_{fn})^{2}+(y_{D}-y_{fDn}-u_{Dn}\sin\theta_{fn})^{2}}\right]$ (D-15)

1. ^*^ Corresponding author, Email: wanglei1986sp@foxmail.com [↑](#footnote-ref-2)
